# Quantitative analysis of the effect of docetaxel-induced edema on quality of life in patients with breast cancer and related factors: a prospective cohort study

**DOI:** 10.1186/s12905-024-03003-4

**Published:** 2024-03-07

**Authors:** Tomoko Izawa, Ami Kobayashi, Masahiro Kawashima, Nobuko Kawaguchi-Sakita, Akiyoshi Nakakura, Yuki Kataoka, Kenichiro Shide, Yukiko Mori, Kazuhiro Yamazaki, Masakazu Toi, Harue Arao

**Affiliations:** 1 Department of Nursing, Kyoto University Hospital, 54 Shogoin-Kawahara-Cho, Sakyo-Ku, Kyoto, 606-8507 Japan; 2 Department of Metabolism and Clinical Nutrition, Kyoto University Hospital, 54 Shogoin-Kawahara-Cho, Sakyo-Ku, Kyoto, 606-8507 Japan; 3 Department of Breast Surgery, Graduate School of Medicine, Kyoto University, 54 Shogoin-Kawahara-Cho, Sakyo-Ku, Kyoto, 606-8507 Japan; 4 Department of Clinical Oncology, Graduate School of Medicine, Kyoto University, 54 Shogoin-Kawahara-Cho, Sakyo-Ku, Kyoto, 606-8507 Japan; 5 Department of Biomedical Statistics and Bioinformatics, Graduate School of Medicine, Kyoto University, 54 Shogoin-Kawahara-Cho, Sakyo-Ku, Kyoto, 606-8507 Japan; 6 Department of Internal Medicine, Kyoto Min-Iren Asukai Hospital, Kyoto, Japan; 7 Scientific Research Works Peer Support Group, Osaka, Japan; 8 Section of Clinical Epidemiology, Department of Community Medicine, Kyoto University Graduate School of Medicine, Kyoto, Japan; 9 Department of Healthcare Epidemiology, Kyoto University Graduate School of Medicine/School of Public Health, Kyoto, Japan; 10 Department of Medical Informatics, Kyoto University Hospital, 54 Shogoin-Kawahara-Cho, Sakyo-Ku, Kyoto, 606-8507 Japan; 11 Department of Cardiovascular Surgery, Graduate School of Medicine, Kyoto University, Japan.54 Shogoin-Kawahara-Cho, Sakyo-Ku, KyotoKyoto, 606-8507 Japan; 12 Tokyo Metropolitan Cancer and Infectious Disease Center, Komagome Hospital, 3-18-22 Honkomagome, Bunkyo-Ku, Tokyo, 113-8677 Japan; 13 Division of Health Science, Graduate School of Medicine, Osaka University, 1-7 Yamadaoka Suita, Osaka, 565-0871 Japan

**Keywords:** Breast cancer, Bioimpedance analysis, Docetaxel, Quality of life, Systemic edema

## Abstract

**Background:**

Systemic edema is an adverse effect of docetaxel chemotherapy and causes distress to patients, including those receiving this agent for breast cancer. However, its characteristics and factors related to its effect on quality of life (QoL) have not been adequately investigated. In this study, we assessed systemic edema quantitatively, explored related factors, and evaluated QoL in patients receiving docetaxel for breast cancer.

**Methods:**

The study had a prospective cohort design and included 37 patients with no known history of swelling who were treated with docetaxel between September 2019 and April 2022. Patients were examined at the start, middle, and end of their course of treatment and 1 and 2 months later. Body water content, body mass, fat mass, and muscle mass were quantified using bioelectrical impedance analysis. Systemic edema was evaluated with reference to the Common Terminology Criteria for Adverse Events. The timing of development of systemic edema at any anatomical site that was grade 2 or worse was recorded. QoL was assessed using the Quality of Life-Anti Cancer Drug scale. Nutrition was evaluated using the Brief-type self-administered diet history questionnaire. Multivariable logistic regression analysis was performed to identify related factors. QoL was also compared between patients with edema and those without edema.

**Results:**

Systemic edema developed in 67% of the study participants and was most prevalent at the end of treatment. Body fat mass (adjusted odds ratio [aOR] 0.802, 95% confidence interval [CI] 0.651–0.988, *p* = 0.038), disease stage (aOR 3.279, 95% CI 0.493–21.793, *p* = 0.219), and history of alcohol consumption (aOR 0.141, 95% CI 0.013–1.521, *p* = 0.106) were identified as risk factors for docetaxel-induced edema. Participants who developed systemic edema experienced more physical, vital, and emotional distress 1 month after treatment than those who did not. There was no association between systemic edema and nutrition.

**Conclusions:**

Systemic edema may develop after treatment with docetaxel and increase distress in patients with a high body fat mass. Patients at risk of systemic edema should be informed in advance about the potential frequency, location, and timing of its onset and encouraged to self-manage this condition.

**Supplementary Information:**

The online version contains supplementary material available at 10.1186/s12905-024-03003-4.

## Background

Paclitaxel and docetaxel belong to the microtubule inhibitor class of cytotoxic anti-cancer drugs and are key agents in the treatment of breast cancer. It is known that women with early-stage breast cancer who are at high risk of recurrence and undergo preoperative or postoperative chemotherapy using a combination of an anthracycline and a taxane are at lower risk of recurrence and survive for longer [1, 2]. Therefore, taxanes are used to treat breast cancer in all stages [1–5].

One of the adverse effects of docetaxel is systemic edema [5–8]. In previous studies, the prevalence of docetaxel-induced edema has ranged from 31.7% to 51% [3, 4, 9–14], being highest when docetaxel was administered in the adjuvant setting. Although edema is well documented in patients with conditions such as heart disease, kidney disease, and anemia [15], its association with docetaxel chemotherapy has not been adequately investigated. Most of the relevant studies have focused on upper extremity lymphedema [11–14], and few have characterized systemic edema, including its timing of onset in relation to the start of treatment and the site(s) affected.

Furthermore, patients undergoing chemotherapy may develop impaired taste and changes in their physical status that affect their food intake [16]. Patients receiving docetaxel for breast cancer have been reported to experience taste disorders and loss of appetite [17]. Such patients often prefer highly seasoned, salt-containing meals, which contribute to development of edema. However, it is not known whether such changes in diet are involved in development of edema because the relationship between diet and systemic edema has not been investigated.

Patients receiving docetaxel can also experience other adverse effects, such as fatigue, numbness, and hair loss, in addition to systemic edema [10–14, 18–21]. Moreover, previous studies have found that quality of life (QoL) is diminished in patients receiving docetaxel because of peripheral neuropathy and fatigue [18–21]. However, the effect of systemic edema on QoL during the course of treatment with docetaxel is poorly understood.

The aim of this study was to ascertain the relationship between systemic edema and associated factors and its impact on QoL in patients receiving treatment with docetaxel for breast cancer. Our hope is that the findings of this research will help to identify patients at high risk for systemic edema and assist with development of supportive care options.

## Methods

### Study design and participants

This prospective cohort study included women treated with docetaxel for breast cancer at the Department of Breast Surgery, Kyoto University Hospital, from September 2019 to April 2022. Women were eligible for inclusion if they were over 20 years of age, had no known history of edema, and were deemed suitable for participation by their attending physician. Enrollment required the absence of edema at the start of treatment; thus, patients with renal disease, cardiac disease, hypertension, or those who had previously received anthracycline-based chemotherapy were also included. Patients were excluded if judged unsuitable by their physician or if they had a known history of edema. All participants received dexamethasone as premedication.

### Ethical approval

The study was approved by Kyoto university graduate school and faculty of medicine, Ethics Committee. (approval number C1438-3). All study participants provided informed consent after receiving a detailed explanation of the aims of the research and the procedures involved.

### Procedures

**Fig. 1:**
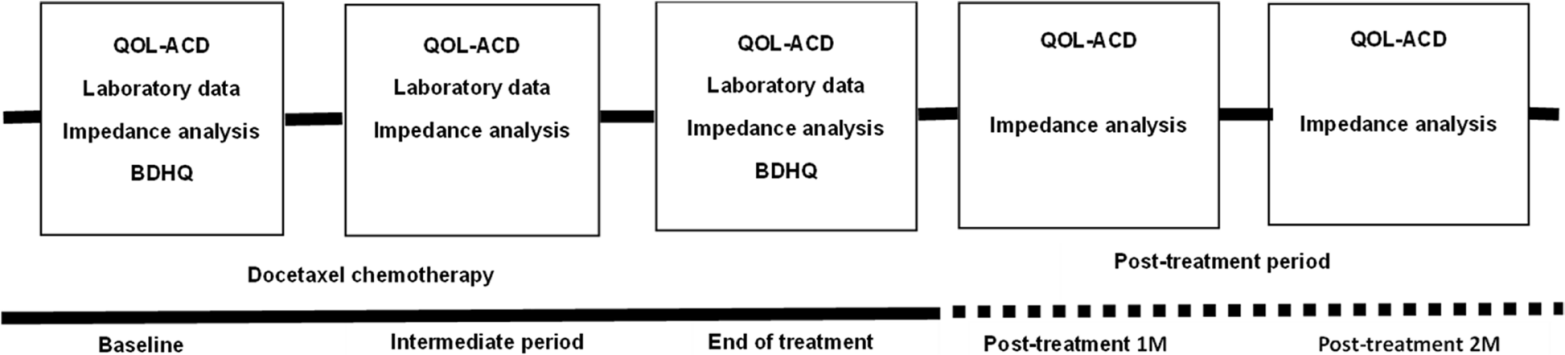
Study design. QOL-ACD: Quality of Life-Anti Cancer Drug, BDHQ: Brief-type self-administered Diet History Questionnaire

The participants were surveyed at five time points: the start of treatment with docetaxel, during treatment, at the end of treatment, and 1 and 2 months following the completion of treatment. Body water content, body mass, body fat mass, and muscle mass were measured using the bioimpedance analysis (BIA) method. This method calculates components of body composition, such as muscle mass and body fat mass, by applying a minor electric current to the body and measuring the resulting electrical resistance, thereby exploiting the body’s conductive properties. Laboratory data were collected, and participants completed QoL questionnaires. A dietitian conducted a nutrition survey at the start and again at the end of treatment. Figure 1 shows the time course of the study.

### Demographic information

The following information was recorded: age, disease stage, chemotherapy regimen, disease subtype, marital status, employment status, educational background, financial status, history of alcohol consumption, and smoking status. Diuretics were administered at the discretion of the physician during chemotherapy, and their administration was recorded.

### Evaluation of systemic edema

No standardized grading system currently exists for quantitative evaluation of systemic edema. Therefore, we have established our own methodology based on the rate of increase in water content, guided by Common Terminology Criteria for Adverse Events (CTCAE) version 5.0 for “separate limb edema” [22]. We calculated the percentage increase in water content at consistent sites (affected arm, unaffected arm, affected leg, unaffected leg, and trunk) between the start of treatment and 2 months after completion of treatment. These values were then graded in accordance with the “separate limb edema” criteria specified in CTCAE version 5.0 [22]. For patients with bilateral breast cancer, the arm corresponding to the breast being treated in the current study cycle was considered to be the affected arm. We categorized a baseline water content increase of ≤ 5% as grade 0, an increase of 5%–10% as grade 1, an increase of 10%–30% as grade 2, and an increase of > 30% as grade 3. Systemic edema was diagnosed when a participant was found to have a score of grade ≥ 2 for any body component. Subsequently, the presence of edema was dichotomized into either existing or not, following assessment of water content in each body component.

### Quality of Life Questionnaire for patients undergoing treatment with Anti-Cancer Drug

QoL was assessed using the Quality of Life-Anti Cancer Drug (QOL-ACD) scale, which was developed by Kurihara et al. as a cancer-specific instrument tailored to Japanese culture and customs [23]. The scale has five subscales: vitality (six items); physical aspect (five items); mental aspect (five items); social aspect (five items); and overall QoL. The design of the subscales takes clinical validity into account. Respondents rate each item on a 5-point scale, 1 being the worst and 5 being the best. The total score on this scale ranges from 22 to 110. Scoring can be done both for individual subscales and for the total aggregated score [24].

### Body mass and composition

Body mass and composition were assessed using the BIA method and a body composition analyzer (Inbody 770; InBody Japan Co, Ltd., Tokyo, Japan). This device differentiates between extracellular and intracellular body water, allowing determination of normative values for total body water. The total body water content (normal range 26.3–32.1 L) and the water content of each body component (arm, normal range 1.18–1.78 L; leg, normal range 4.21–5.15 L; trunk, normal range 12.1–14.8 L) were measured. Body mass index (BMI) was calculated using patient height and weight. Body fat and muscle mass have been examined previously [21, 25]. In the present study, we measured body fat mass (normal range 10.1–16.1 kg) and muscle mass (normal range 33.0–40.4 kg). These variables were treated as continuous, with no specific cutoff values established. Participants were asked to stand on the Inbody 770 machine while barefoot for 1 min to measure their body composition before starting treatment with docetaxel.

### Laboratory data

Laboratory data were obtained in accordance with routine medical practice. Values for several biomarkers, including total protein, albumin, hemoglobin, blood urea nitrogen, creatinine clearance, and zinc, were used to assess nutritional status and systemic edema before administration of docetaxel. These values were analyzed as continuous variables, with no specific cutoff values established.

### Brief-type self-administered diet history questionnaire

Diet was evaluated using the Japanese version of the Brief-type self-administered diet history questionnaire (BDHQ). This instrument had been validated by calculating the consumption of approximately 30 nutrients and 50 foods using a nutritional value formula [26]. Energy, carbohydrate, protein, fat, zinc, and sodium intake were evaluated.

### Sample size

Based on the reported incidence of edema in patients with breast cancer receiving docetaxel [9], the null hypothesis was set at 0.47 and the alternative hypothesis was set at 0.25 if nutritional guidance was included. Using a one-sided alpha value of 0.05, a power of 0.8, an anticipated dropout rate of 0.2, and a binomial test, the required number of participants was calculated to be 33.

### Statistical analysis

We calculated descriptive statistics for participants who had data available for at least three of the five survey time points. Systemic edema was determined using the CTCAE grading system and treated as a binary outcome. We then compared the demographic and clinical characteristics according to edema status. Differences in age, body mass, body fat mass, BMI, disease stage and subtype, smoking and alcohol consumption, diuretic use, marital and educational status, and income were examined using the chi-squared test for categorical variables and the Mann–Whitney *U* test for continuous variables.

To assess the impact on patient QoL, we compared each QoL subscale score between participants with and without edema at all time points from initiation of docetaxel to 2 months after completion of treatment using the Student’s* t*-test. Complete case analyses were then performed, excluding participants who reported awareness of swelling at the time of enrollment in the study. All statistical analyses were performed using SPSS version 27.0 (IBM Corp., Armonk, NY, USA). A two-sided p-value of < 0.05 was considered statistically significant. No correction was made for multiplicity.

A further analysis was performed to identify factors associated with systemic edema. Potential predictors, including age, disease stage, treatment objective, history of smoking and alcohol consumption, QoL subscale scores, body mass, body fat, muscle mass, BMI, laboratory data, and nutrient intake, were sought in univariate logistic regression analysis. These continuous variables were accounted for at baseline. Variables with a p-value of < 0.2 and those deemed to have a plausible association with systemic edema in univariate analysis were entered into a multivariable logistic regression model.

## Results

### Patients

**Fig. 2:**
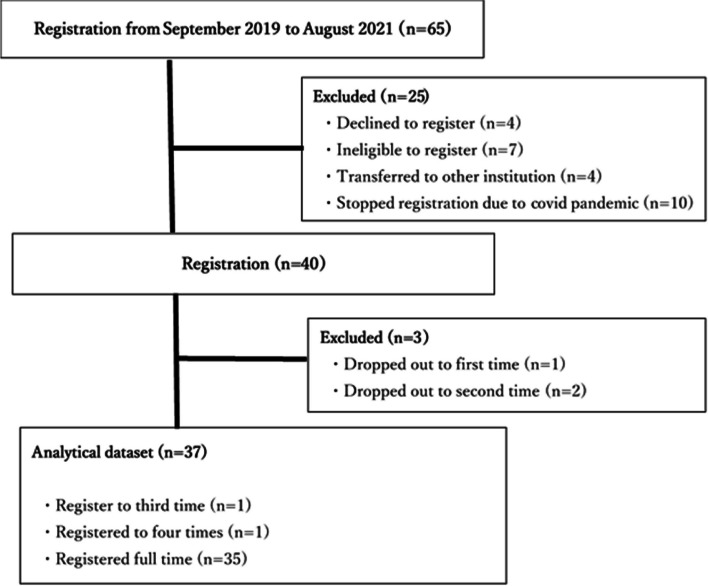
Flow diagram of participation in the study

During the study period, 65 patients received docetaxel 75 mg/m^2^ for 4–8 cycles. Four of these patients declined to participate in the study, three discontinued participation after the first or second visit, four were transferred to other institutions, seven fulfilled one of the exclusion criteria, and 10 could not be recruited because of the coronavirus pandemic. Therefore, 37 patients participated in the study; one only participated up to the end of treatment and another only participated for up to 1 month after completion of treatment because they were referred to another institution for radiotherapy. Figure 2 shows the flow of patients through the study.

The patient background characteristics are summarized in Table 1. The median age was 52 years (interquartile range [IQR] 44, 61). The breast cancer was unilateral in 30 patients (81%) and bilateral in seven (19%). The most common disease stage was II (*n* = 18, 49%). Fifteen participants (41%) received four cycles of docetaxel as neoadjuvant chemotherapy, 15 (41%) received four cycles as adjuvant therapy, and seven (19%) received 5–8 cycles for metastatic disease. Seventeen patients (46%) received docetaxel 75 mg/m^2^ in combination with trastuzumab and pertuzumab and 15 (41%) received docetaxel 75 mg/m^2^ alone. The patients who received docetaxel as neoadjuvant or adjuvant therapy had previously undergone four cycles of chemotherapy with doxorubicin hydrochloride and cyclophosphamide. The most common subtypes of breast cancer were luminal and luminal HER2, with 17 patients (46%) having each subtype. Eighteen patients (49%) were premenopausal and 19 (51%) were postmenopausal. Eleven (29%) had a history of alcohol consumption. Seven (18%) were prescribed a diuretic (furosemide 10 mg or 20 mg) during the study period; in all instances, the diuretic was prescribed during the first month after completion of treatment with docetaxel. No participant in the study developed cardiac or renal insufficiency after starting treatment with docetaxel.

**Table 1:** Background characteristics of the study participants (*n* = 37)

		**Total *n* = 37**	
		Median	IQR
**Age (years)**		52	44–61
**Body mass at baseline (kg)**		53.6	49.1–58.2
**Body fat mass at baseline (kg)**		15.3	11.8–19.2
**BMI at baseline (kg/m**^**2**^**)**		21.7	19.2–23.3
		**n**	**%**
**Disease**	Right breast cancer	18	48.6
	Left breast cancer	12	32.4
	Bilateral breast cancer	7	18.9
**Purpose of the treatment**	Neoadjuvant	15	40.5
	Adjuvant	15	40.5
	Recurrence or advanced	7	18.9
**Disease stage**	I	7	18.9
	II	18	48.6
	III	5	13.5
	IV	7	18.9
**Disease subtype**	ER + /Her2 −	17	45.9
	ER − /Her2 +	3	8.1
	ER + /Her2 +	17	45.9
**Chemotherapy regimen**	T	15	40.5
	THP	17	45.9
	TCbHP	3	8.1
	TC	2	5.4
**Her2 status**	Her2 +	20	54.1
	Her2 −	17	45.9
**Menopausal status**	Pre-menopausal	18	48.6
	Post-menopausal	19	51.4
**Smoker**	Yes	8	21.6
	No	29	78.4
**History of alcohol consumption**	Yes	11	29.7
	No	26	70.3
**Use of diuretics**	Yes	7	18.9
	No	30	81.1
**Marital status**	Yes	19	51.4
	No	18	48.6
**In employment**	Yes	15	40.5
	No	22	59.5
**Educational background**	Junior high school	2	5.4
	High school	9	24.3
	College school	8	21.6
	Undergraduate or above	17	45.9
	No answer	1	2.7
**Income**	< 2 million yen p.a	4	10.8
	2–6 million yen p.a	13	35.1
	> 6 million yen p.a	7	18.9
	No answer	13	35.1

*BMI* Body mass index, *ER* Estrogen receptor, *HER2* Human epidermal growth factor receptor 2, *IQR* Interquartile range, *p.a.* per annum, *T* Docetaxel, *TC* Docetaxel and cyclophosphamide, *TCbHP* Docetaxel, carboplatin, trastuzumab, and pertuzumab, *THP* Docetaxel, trastuzumab, and pertuzumab

### Characterization of systemic edema according to site, severity, and time of onset

**Fig. 3:**
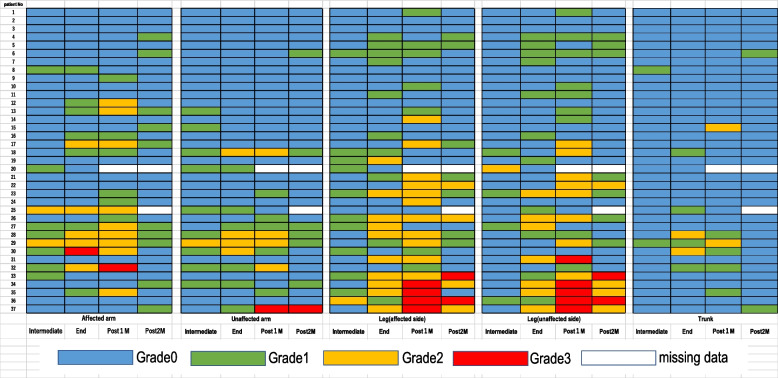
Characteristics of systemic edema (site and timing). The grade of edema is indicated by color, with edema less than Grade 1 being shown as Grade 0 in blue, Grade 1 in green, Grade 2 in orange, and Grade 3 in red. The figure shows the severity of edema in the affected arm, unaffected arm, affected leg, unaffected leg, and trunk

Figure 3 shows the characteristics of the systemic edema in the 37 study participants. The arms are divided into affected or unaffected sides depending on the location of the breast cancer. Grade 2 edema was more common in the affected arm (24%) than in the unaffected arm (11%) at 1 month after treatment. Systemic edema was more commonly observed in the legs than in the arms. One month after completion of treatment, we observed grade 2 leg edema in 22%–30% of cases and grade 2 arm edema in 11%–24%. Grade 3 leg edema was present in 11%–14% of cases and grade 3 arm edema in 3%. Patient 37 appeared to have much worse edema in their unaffected arm than in their affected arm. This participant had a history of breast cancer in the contralateral breast and had undergone lymph node dissection in the unaffected arm.

Systemic edema occurred in four patients (11%) during treatment and in 14 (38%) at the end of treatment. Seven (19%) still had systemic edema at the end of the first month after completion of treatment. Twelve (32%) of the patients did not develop systemic edema during the study period. Overall, systemic edema developed in 25 (67%) of our study participants.

### Relationship between systemic edema and QoL

**Fig. 4:**
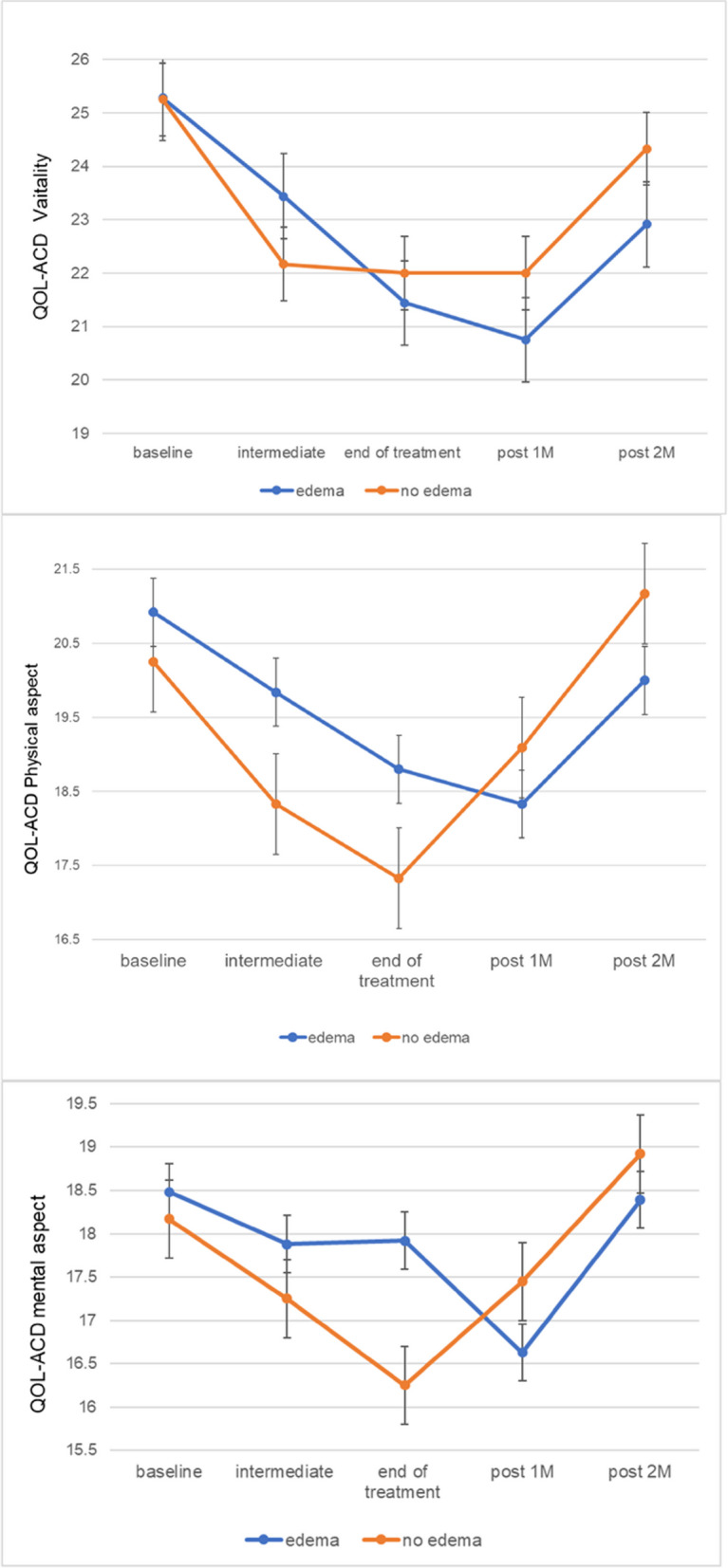
Longitudinal changes in QOL-ACD and systemic edema

Table 2 showed the relationship between systemic edema and QoL during the time course of the study. Figure 4 shows the QoL subscale scores according to edema status. There was no statistically significant difference between-group in the vitality or physical, mental, and social aspect subscale scores or in the total QoL score at any time point in the study. Notably, at 1-month post-treatment, participants with edema recorded the lowest (albeit not statistically significant) mean subscale scores for vitality (20.8 [SD 5.4] for the edema group vs. 22 [5.1] for the non-edema group), physical aspect (18.3 [SD 4.3] vs. 19.1 [4.1]), and mental aspect (16.6 [SD 4.6] vs. 17.5 [SD 4.2]). By 2 months after treatment, scores for those with edema had somewhat improved but continued to show a consistent trend of being lower than in those without edema.

**Table 2:** Relationship between systemic edema and quality of life score at five assessment time points

	**Vitality**		**Physical aspect**		**Mental aspect**		**Social aspect**		**Total QOL score**	
	**Mean (SD)**	***P-value***	**Mean (SD)**	***P-value***	**Mean (SD)**	***P-value***	**Mean (SD)**	***P-value***	**Mean (SD)**	***P-value***
**Baseline**										**-**
**edema**	25.28(4.72)	0.987	20.92(3.50)	0.582	18.48(4.31)	0.831	13.96(3.86)	0.718	83.32(15.36)	0.626
**no edema**	25.25(5.63)		20.25(3.28)		18.17(3.81)		13.42(5.02)		80.67(15.32)	
**Intermediate period**										
**edema**	23.44(5.54)	0.487	19.84(3.59)	0.216	17.88(3.78)	0.621	15.28(3.45)	0.431	81.12(13.66)	0.214
**no edema**	22.17(4.22)		18.33(2.96)		17.25(3.17)		14.17(4.95)		75.25(12.20)	
**End of treatment**										
**edema**	21.44(5.29)	0.772	18.80(3.81)	0.293	17.92(4.24)	0.259	14.92(4.17)	0.631	77.68(15.46)	0.398
**no edema**	22.00(5.83)		17.33(4.14)		16.25(3.93)		14.17(4.93)		72.92(16.70)	
**Post-treatment 1 M**										
**edema**	20.75(5.35)	0.521	18.33(4.26)	0.625	16.63(4.60)	0.615	15.04(3.51)	0.888	75.17(13.35)	0.677
**no edema**	22.00(5.14)		19.09(4.11)		17.45(4.20)		15.27(6.13)		77.36(16.46)	
**Post-treatment 2 M**										
**edema**	22.91(5.46)	0.480	20.00(2.86)	0.306	18.39(3.49)	0.709	14.83(3.50)	0.768	80.13(13.29)	0.643
**no edema**	24.33(5.81)		21.17(3.66)		18.92(4.66)		14.33(6.37)		82.58(17.29)	

Differences between the two groups were compared using the Student’s* t*-test

*1 M* 1 month, *2 M* 2 months, *SD* Standard deviation

### Factors related to systemic edema

Table 3 presents the patient characteristics according to whether or not they developed edema. There were statistically significant differences between the group with edema and the group without edema in terms of median body mass (55.5 [IQR 44.5, 64.0] vs 50.6 [IQR 46.0, 53.4], *p* = 0.017), body fat mass (17.3 [IQR 12.7, 20.3] vs 12.3 [IQR 10.9, 15.5], *p* = 0.019), and BMI (22.7 [IQR 19.9, 23.8] vs 19.6 [IQR 18.9, 22.3], *p* = 0.014). There was also a significant between-group difference in history of alcohol consumption (*p* = 0.049).

**Table 3:** Background characteristics according to whether or not participants developed docetaxel-induced systemic edema

		**edema *n* = 25**		**no edema *n* = 12**		***P-value***
		Median	IQR	Median	IQR	
**Age (years)**		54	44.5–64	48	43.5–51.8	0.109
**Body mass at baseline (kg)**		55.5	52.2–59	50.6	46.0–53.4	0.017*
**Body fat mass at baseline (kg)**		17.3	12.7–20.3	12.3	10.9–15.5	0.019*
**BMI at baseline (kg/m**^**2**^**)**		22.7	19.9–23.8	19.6	18.9–22.3	0.014*
		**n**	**%**	**n**	**%**	
**Disease**	Right breast cancer	10	40.0	8	66.6	0.103
	Left breast cancer	8	32.0	4	33.4	
	Bilateral breast cancer	7	28.0	0	0	
**Purpose of the treatment**	Neoadjuvant	9	36.0	6	50.0	0.484
	Adjuvant	10	40.0	5	41.6	
	Recurrence or advanced	6	24.0	1	8.3	
**Disease stage**	I	5	20.0	2	16.6	0.451
	II	10	40.0	8	66.6	
	III	4	16.0	1	8.4	
	IV	6	24.0	1	8.4	
**Disease subtype**	ER + /Her2 −	13	52.0	4	33.4	0.156
	ER − /Her2 +	3	12.0	0	0	
	ER + /Her2 +	9	36.0	8	66.6	
**Chemotherapy regimen**	T	11	44.0	4	33.3	0.619
	THP	10	40.0	7	58.3	
	TCbHP	2	8.0	1	8.4	
	TC	2	8.0	0	0	
**Her2 status**	Her2 +	12	48.0	8	66.7	0.286
	Her2 −	13	52.0	4	33.3	
**Menopausal status**	Pre-menopausal	11	44.0	7	58.3	0.414
	Post-menopausal	14	56.0	5	41.7	
**Smoker**	Yes	4	16.0	4	33.3	0.231
	No	21	84.0	8	66.7	
**History of alcohol consumption**	Yes	10	40.0	1	16.7	0.049*
	No	15	60.0	11	83.3	
**Use of diuretics**	Yes	5	20.0	2	16.7	0.809
	No	20	80.0	10	83.3	
**Marital status**	Yes	13	52.0	6	50.0	0.909
	No	12	48.0	6	50.0	
**In employment**	Yes	10	40.0	5	41.7	0.923
	No	15	60.0	7	58.3	
**Educational background**	Junior high school	1	4.0	1	8.4	0.506
	High school	8	32.0	1	8.4	
	College school	5	20.0	3	25.0	
	Undergraduate or above	10	40.0	7	58.3	
	No answer	1	4.0	0	0	
**Income**	< 2 million yen p.a	2	8.0	2	16.6	0.171
	2–6 million yen p.a	8	32.0	5	41.7	
	> 6 million yen p.a	3	12.0	4	33.3	
	No answer	12	48.0	1	8.4	

^*^p < 0.05, Pearson’s chi-squared test or Mann–Whitney *U* test

*BMI* Body mass index, *ER* Estrogen receptor, *HER2* Human epidermal growth factor receptor 2, *IQR* Interquartile range, *p.a.* per annum, *T* Docetaxel, *TC* Docetaxel and cyclophosphamide, *TCbHP* Docetaxel, carboplatin, trastuzumab, and pertuzumab, *THP* Docetaxel, trastuzumab, and pertuzumab

Table 4 showed the findings of the univariate and multivariable logistic regression analyses. In univariate analysis, significant associations were found for body fat mass (odds ratio [OR] 1.217, 95% confidence interval [CI] 1.006–1.472, *p* = 0.044), blood urea nitrogen level (OR 1.345, 95% CI 1.001–1.808, *p* = 0.049), and BMI (OR 1.388, 95% CI 0.997–1.932, *p* = 0.052). Multivariable logistic regression analysis identified increased body fat mass (adjusted OR [aOR] 0.802, 95% CI 0.651–0.988, *p* = 0.038), disease stage (aOR 3.279, 95% CI 0.493–21.793, *p* = 0.219), and a history of alcohol consumption (aOR 0.141, 95% CI 0.013–1.521, *p* = 0.106) to be associated factors, although the latter two variables did not reach statistical significance.

**Table 4:** Results of multivariate logistic analysis of potential risk factors for systemic edema

**Variable name**	**Full model**			**Reduced model**		
	**Cured OR**	**95% CI**	***P-*value**	**Adjusted OR**	**95% CI**	***P*-value**
**Age(years)**						
**65 < **	0.633	0.107–3.733	0.614			
**65 > **	**Ref**					
**Disease stage**						
I-II	0.300	0.054–1.669	0.169	3.279	0.493–21.793	0.219
III-IV	**Ref**					
**Menopausal status**						
Premenopausal	0.561	0.139–2.260	0.416			
Post menopausal	**Ref**					
**Her2-status**						
Her2-positive	0.462	0.110–1.936	0.291			
Her2-negative	**Ref**					
**Purpose of the treatment**						
Neoadjuvant	0.250	0.024–2.636	0.249			
Adjuvant	0.333	0.031–3.579	0.364			
Recurrence/Advance	**Ref**					
**Smoker**						
Yes	0.381	0.076–1.901	0.239			
No	**Ref**					
**History of alcohol consumption**						
Yes	0.193	0.021–1.766	0.145	0.141	0.013–1.521	0.106
No	**Ref**					
**Use of diuretics**						
Yes	0.800	0.131–4.874	0.809			
No	**Ref**					
**Body mass**	1.126	0.996–1.273	0.058			
**Body fat mass**	1.217	1.006–1.472	0.044	0.802	0.651–0.988	0.038
**Muscle mass**	1.147	0.935–1.406	0.188			
**BMI**	1.388	0.997–1.932	0.052			
**TP**	1.398	0.368–5.310	0.623			
**Alb**	1.129	0.217–5.867	0.885			
**Hb**	1.452	0.935–2.256	0.097			
**Zn**	1.019	0.957–1.086	0.559			
**BUN**	1.345	1.001–1.808	0.049			
**Energy intake**	1	0.998–1.002	0.930			
**Protein intake**	1.020	0.974–1.068	0.407			
**Carbohydrate intake**	0.997	0.985–1.008	0.566			
**Fat intake**	0.995	0.950–1.042	0.832			
**Zn intake**	1.070	0.724–1.581	0.733			
**Salt equivalent intake**	1.019	0.784–1.325	0.888			
**QOL-ACD vitality**	1.001	0.870–1.153	0.986			
**QOL-ACD physical aspect**	1.060	0.866–1.297	0.572			
**QOL-ACD mental aspect**	1.019	0.860–1.208	0.826			
**QOL-ACD social aspect**	1.032	0.873–1.221	0.710			

*Alb* Albumin, *BMI* Body mass index, *BUN* Blood urea nitrogen, *CI* Confidence interval, *Hb* Hemoglobin, *HER2* Human epidermal growth factor receptor 2, *OR* Odds ratio, *QOL-ACD* Quality of Life-Anti Cancer Drug, *TP* Total protein, *Zn* Zinc

## Discussion

In this study, 67% of patients who received docetaxel developed systemic edema, which peaked at the conclusion of treatment. We also observed that patients with edema had lower QoL scores, particularly on the vitality and the physical and mental aspect subscales and recovered more slowly than those without edema at 1 month after completion of treatment. Furthermore, an association was found between increased body fat mass and development of edema.

The overall prevalence of systemic edema was 67% in our study, which is higher than that previously reported. One study that compared docetaxel + trastuzumab with vinorelbine + trastuzumab in HER2-positive breast cancer reported that the incidence of edema was 31.7% in the docetaxel group and only 3.6% in the vinorelbine group [3]. In another study, 51% of patients receiving ongoing treatment with docetaxel 60 mg/m2 for recurrent breast cancer developed systemic edema after 10 cycles [10]. Although BIA has been used in earlier studies to assess the severity of upper extremity lymphedema, it is not accurate enough for assessment of systemic edema [25]. The findings of the present study are important because we calculated the precise water content for various body components and accurately determined the prevalence of systemic edema. Notably, although systemic edema was most prevalent at the end of treatment, in some cases it developed as late as 1 month after completion of treatment, and with a higher prevalence of grade 3 edema at that time point. Development of systemic edema has been linked to a cumulative docetaxel dose of 400 mg/m2 [6]. However, we found that some patients developed grade 2 edema after only four cycles of docetaxel at a dose of 75 mg/m2, which amounts to a total of 300 mg/m2 and is lower than that previously reported. The distribution of systemic edema usually involves the extremities and trunk, with a higher frequency in the lower legs and the arm affected by previous lymph node dissection [11–14]. In line with a previous finding that edema frequently affects the lower legs in patients treated with docetaxel for recurrent breast cancer [10], this study demonstrated that systemic edema mainly involves excessive water retention in the lower legs, in which the storage capacity is greater than that in the upper extremities. In this regard, our study provides a comprehensive picture of the prevalence and characteristics of docetaxel-induced systemic edema.

Participants who developed systemic edema during treatment with docetaxel were observed to have reduced QoL at 1 month after completion of treatment, suggesting that systemic edema has a significant impact on patients’ well-being. This temporal association was highlighted by the decreases in QoL subscale scores coinciding with the development of systemic edema, indicating that edema may be associated with increased physical and psychological distress as well as diminished vitality. This finding is consistent with previous research showing that edema is associated with subjective distress during chemotherapy and for up to 6 months after its completion and that the edema caused by the docetaxel regimen results in greater distress than that associated with other treatments [5]. Although the impact of edema on QoL may be perceived as minor, our findings indicate that it is indeed a troubling symptom. Current supportive measures for patients receiving docetaxel therapy focus primarily on peripheral neuropathy, skin disorders, and hematological toxicity [18–20]. However, it is essential that health care providers also address the distress caused by systemic edema and develop appropriate supportive strategies.

Our research determined an association between systemic edema and body fat mass. Previous studies have established a link between the etiology of upper limb lymphedema and BMI [13, 14, 25]. In our present study, we found that body fat mass was correlated with not only lymphedema in the affected arm but also systemic edema. The principal mechanism underlying docetaxel-induced edema is believed to be increased capillary permeability. Development of edema has been reported to be dose-dependent, presumably owing to a reduction in interstitial fluid pressure, which leads to accumulation of interstitial fluid and compromised lymphatic return [6]. Individuals with a high amount of body fat may have a larger number of adipocytes in the interstitial space, potentially exacerbating the impaired return of fluid to the lymphatic system. Furthermore, disease stage was found to contribute to systemic edema. Additional analysis of the association between breast cancer stage and edema development is needed. We also observed a tendency for edema to develop in patients with a history of alcohol consumption. Alcohol consumption during treatment might further leak fluid out of the blood vessels. However, diet, as assessed by the BDHQ, did not show a definitive correlation with systemic edema. Although docetaxel-induced alterations in taste have been reported [17], they may not be pronounced enough to affect nutritional status and induce systemic edema. Further studies examining the association of docetaxel-induced changes in taste with nutrient intake are needed.

We also found that docetaxel-induced systemic edema often develops after completion of treatment and frequently presents specific challenges in the legs, which has important implications for clinical practice. It is essential for health care providers to inform patients before initiation of treatment with docetaxel about the potential for systemic edema, which, according to our data occurs in approximately two-thirds of patients. In particular, patients with a higher amount of body fat should be forewarned about the risk of leg-dominant edema and should receive guidance on self-management techniques. Health care providers must remain vigilant for the possible onset of this adverse effect during and following chemotherapy to alleviate the impact of edema on patients’ ability to perform basic activities of daily living.

This study has several limitations. First, being conducted at a single institution, the findings may only reflect the unique characteristics of that specific setting. Second, the relatively small sample size could have hindered our ability to understand the etiology of docetaxel-induced edema and its impact on QoL in detail. Third, the possibility of selection bias cannot be excluded, notably the potential underrepresentation of patients with more severe edema or other adverse effects. Finally, the study focused on docetaxel-induced systemic edema in a cohort of Japanese patients with breast cancer. Whether these findings can be extrapolated to non-Asian populations or to cases of edema induced by other anticancer agents remains uncertain. Future research should include such groups to ascertain the generalizability of our results.

In conclusion, systemic edema was observed in 67% of patients treated with docetaxel for breast cancer between initiation of therapy and up to 2 months after treatment, peaking at completion of treatment and affecting predominantly the lower extremities. Physical, vital, and emotional distress 1 month following completion of treatment may be greater in patients who develop systemic edema than in those who do not. An association between body fat mass and systemic edema was identified. Our findings underscore the importance of proactively informing patients about the risk of docetaxel-induced systemic edema, including its potential frequency, anatomic distribution, and temporal pattern, and encouraging them to actively engage in self-management.

### Supplementary Information

**Supplementary Material 1.**

## Data Availability

The full data set analyzed in this study is available from the corresponding author on reasonable request.

## Abbreviations

Alb: Albumin
BDHQ: Brief-type Self-Administered Diet History Questionnaire
BIA: Bioimpedance analysis
BMI: Body mass index
BUN: Blood urea nitrogen
CTCAE: Common Terminology Criteria for Adverse Events
Hb: Hemoglobin
IQR: Interquartile range
QOL-ACD: Quality of Life-Anti Cancer Drug
T: Docetaxel
THP: Docetaxel, trastuzumab, and pertuzumab
TCbHP: Docetaxel, carboplatin, trastuzumab, and pertuzumab
TC: Docetaxel and cyclophosphamide
TP: Total protein
Zn: Zinc
