# Supplementary material for: Quantitative analysis of the effect of docetaxel-induced edema on quality of life in patients with breast cancer and related factors: a prospective cohort study
Source: BMC Womens Health. 2024 Mar 7;24:165. doi: 10.1186/s12905-024-03003-4 (PMC10921572; doi:10.1186/s12905-024-03003-4)
Supplement: Supplementary file 1 — Supplementary Material 1. [file 12905_2024_3003_MOESM1_ESM.docx]

“Quantitative analysis of the effect of docetaxel-induced edema on quality of life in patients with breast cancer and related factors: a prospective cohort study”

***BMC Women’s Health***

Tomoko Izawa, Ami Kobayashi, Masahiro Kawashima, Nobuko Kawaguchi-Sakita, Akiyoshi Nakakura, Yuki Kataoka, Kenichiro Shide, Yukiko Mori, Kazuhiro Yamazaki, Matasazu Toi, Harue Arao

Corresponding author: Tomoko Izawa, RN. Ph.D.

Department of Nursing, Kyoto University hospital, Kyoto, Japan. 54 Shogoin-kawahara-cho, sakyo-ku, Kyoto 606-8507, Japan

[tizawa@kuhp.kyoto-u.ac.jp](mailto:tizawa@kuhp.kyoto-u.ac.jp)

**Online Resource 1. Prevalence of systemic edema according to site and period of the study (n=37)**

|  |  | Intermediate period | End of treatment | Post-treatment 1M | Post-treatment 2M |
| --- | --- | --- | --- | --- | --- |
|  |  | n (%) | n (%) | n (%) | n (%) |
| Affected arm | Grade０ | 28 (75.7%) | 24 (64.9%) | 20 (54.1%) | 25 (67.6%) |
|  | Grade１ | 7 (18.9%) | 7 (18.9%) | 6 (16.2%) | 10 (27%) |
|  | Grade２ | 2 (5.4%) | 5 (13.5%) | 9 (24.3%) | - |
|  | Grade３ | - | 1 (2.7%) | 1 (2.7%) | - |
| Unaffected arm | Grade０ | 27 (73%) | 27 (73%) | 26 (70.3%) | 28 (75.7%) |
|  | Grade１ | 9 (24.3%) | 6 (16.2%) | 5 (13.5%) | 6 (16.2%) |
|  | Grade２ | 1 (2.7%) | 4 (10.8%) | 4 (10.8%) | - |
|  | Grade３ | - | - | 1 (2.7%) | 1 (2.7%) |
| Leg (affected side) | Grade０ | 26 (70.3%) | 17 (45.9%) | 14 (37.8%) | 22 (59.5%) |
|  | Grade１ | 10 (27.0%) | 10 (27.0%) | 7 (18.9%) | 7 (18.9%) |
|  | Grade２ | 1 (2.7%) | 10 (27.0%) | 11 (29.7%) | 4 (10.8%) |
|  | Grade３ | - | - | 4 (10.8%) | 2 (5.4%) |
| Leg (unaffected side) | Grade０ | 32 (86.5%) | 19 (51.4%) | 14 (37.8%) | 22 (59.5%) |
|  | Grade１ | 4 (10.8%) | 11 (29.7%) | 9 (24.3%) | 7 (18.9%) |
|  | Grade２ | 1 (2.7%) | 7 (18.9%) | 8 (21.6%) | 4 (10.8%) |
|  | Grade３ | - | - | 5 (13.5%) | 2 (5.4%) |
| Trunk | Grade０ | 35 (94.6%) | 31 (83.8%) | 30 (81.1%) | 33 (89.2%) |
|  | Grade１ | 2 (5.4%) | 4 (10.8%) | 3 (8.1%) | 2 (5.4%) |
|  | Grade２ | - | 2 (5.4%) | 3 (8.1%) | - |
|  | Grade３ | - | - | - | - |
